# An Effect of Smelled Breast Milk During and After Venous Blood Drawing on Newborn Infants’ Pain and Comfort Level

**DOI:** 10.3390/healthcare13162005

**Published:** 2025-08-14

**Authors:** Feyza Kübra Albayram, Selver Guler, Melike Yavas Celik

**Affiliations:** 1 Department of Nursing, SANKO University, Gaziantep 27310, Turkey; feyzakubra_123@hotmail.com; 2 Department of Nursing, Kilis 7 Aralık University, Kilis 79000, Turkey; selver.gulerr@kilis.edu.tr; 3 Department of Midwifery, Gaziantep University, Gaziantep 27310, Turkey

**Keywords:** comfort level, pain, smelling maternal breast milk

## Abstract

**Background/Objectives:** This study was conducted as a randomized controlled trial to evaluate the effect of breast milk odor on pain and stress levels during venous blood sampling. **Method:** Data were collected from 60 newborn infants consisting of a total of 30 infants in the experimental group and 30 in the control group who were hospitalized in the Intensive Care Unit between 7 December 2022 and 25 September 2023. The data were collected using the Newborn Infant Pain Scale and Premature Infant Comfort Scale forms. **Results:** It was observed that there was a statistically significant difference between the groups in terms of the Newborn Infant Pain Scale and Premature Infant Comfort Scale when comparing the measurements taken during the procedure and 5 min after the procedure. A strong correlation was found between the Newborn Infant Pain Scale and Premature Infant Comfort Scale scores measured 5 min after the venous blood collection procedure (r = 0.86, *p* = 0.01). **Conclusions:** Based on the results of this study, it was determined that smelling breast milk, which is one of the non-pharmacological methods that can be used in invasive or non-invasive painful procedures on newborn infants, positively affects the pain and comfort level of babies.

## 1. Introduction

Babies born before 38 weeks of gestation are defined as premature [1]. Since preterm babies are anatomically and physiologically immature, they are usually followed up in the Neonatal Intensive Care Unit (NICU) for diagnosis and treatment, depending on the baby’s needs and health problems. The physical structure of the NICU, the noise of the staff and vehicles used, routine care, and many invasive procedures can affect the pain and comfort levels of babies. For this reason, the recovery and discharge times of newborns may be negatively affected [2].

A baby admitted to the NICU is exposed to many procedures (such as a heel prick or venipuncture, venous or arterial catheterization, chest tube placement, intubation or aspiration, lumbar puncture, subcutaneous or intramuscular injection, surgical interventions, or mechanical ventilation therapy) that cause pain, for diagnosis or treatment purposes [3]. Pain is defined as an unpleasant biochemical condition or experience originating from a specific area of the body, which may or may not be related to tissue damage, and the aim is to eliminate the undesirable situation [4]. It may delay a newborn infant’s adaptation to the outside world. In addition, pain can negatively affect infant behavior, family–infant interactions, growth and development, brain development, and sensory development [5]. The incidence of remembering past pain experiences is higher in premature newborns than in term newborns [6]. In the later stages, recurrent pain in newborns affects the pain threshold, pain perception and pain tolerance, and it may cause hyperalgesia [6].

Behavioral responses that occur during the experience of pain can be exemplified as changes in facial expression, crying, restlessness, moaning, difficulty feeding, excessive extension, a change in tone, alertness, and restlessness [7]. Physiological changes that occur during the occurrence of pain can be listed as an increase in heart rate and blood pressure, a decrease in O_2_ (oxygen) saturation and partial O_2_ pressure, a decrease in vagal tone, and an increase in respiratory rate and intracranial pressure [7]. The biochemical response to pain manifests itself through changes in hormone levels. While catecholamines, cortisol, b-endorphin, growth hormone, glucagon, renin, and aldosterone increase, insulin secretion is mostly suppressed [7].

Premature babies receiving special care in the Neonatal Intensive Care Unit are exposed to many stimuli, such as light and sound, that can negatively impact their health. High-level noise and prolonged exposure to high-voltage lights have been reported to have many negative consequences for premature babies, including tachycardia, tachypnea, restlessness, rapid eye movements, grimacing, crying, startlement, apnea, hypoxia, bradycardia, irregular sleep cycles, an increased intracranial pressure, growth hormone suppression, increased bilirubin levels, changes in the autonomic nervous system, and hypoxic ischemia [8].

Pain perception in the newborn begins in the intrauterine period. Anatomical, neurophysiological, and hormonal components must develop for the fetus to identify pain. Communication between the cortex and thalamus begins after the 20th prenatal week. The fetus begins to sense pain in the second trimester. From birth onward, nociceptors respond to mechanical, thermal, and chemical stimuli, as well as peripheral sensitivity or primary hyperalgesia, and pain signals reach the somatosensory cortex. Nociception begins between the 20th and 22nd weeks [9]. Nociceptors are most densely located on the skin surface, periosteum, arterial walls, and joint surfaces. Babies respond physiologically, behaviorally, and hormonally to pain. In the newborn, the neural pain pathway begins from sensory receptors in the skin and progresses to the cortex in the brain. Memory, perceptual awareness, and consciousness are all involved in this system. However, studies have shown that neonatal nociceptors are denser than in adults. Therefore, newborn babies, especially premature babies, are more sensitive to nociceptive stimuli than older children and adults [9].

Research has shown that non-nutritious sucking reduces behavioral distress and restlessness and appears to modulate the transmission or the process of pain perception by the internal non-opioid system [10]. In a study by Liu et al. on neonatal pain control, infants who were allowed to carry out non-nutritious sucking during painful blood sampling procedures showed less pain responses [11]. Another study by Badiee et al. in 2013, aimed at comparing the effect of breast milk and formula odor on infant relaxation, found that breast milk odor had a pain-reducing effect in infants and could be used as a non-pharmacological pain reduction method [12]. Breastfeeding, breast milk smell, and breast milk taste interventions had large effect sizes for pain management during and after the procedures. These interventions also had medium effect sizes for heart rate during the procedures and large effect sizes after the procedures. They also had large effect sizes for oxygen saturation during the procedures and medium effect sizes after the procedures [13].

In a study conducted using breast milk sniffing, which is one of these sensory stimuli, 48 healthy term babies were examined, and the babies were made to smell their own mother’s milk, another mother’s milk and the smell of formula milk during the heel prick procedure. The pain status of the baby was examined. Babies who smell their own mother’s breast milk have been found to feel less pain and are calmer [14]. In addition, another study reported that the most effective method for reducing pain in newborns was the smell of their own mother’s milk, but the smell of another mother’s milk could also be used for newborns who cannot access their own mother’s milk [15].

Non-pharmacological methods are of great importance due to the negative effects of pharmacological treatments, which are considered the gold standard in pain management [14]. In reducing pain in newborns, many senses can be used to divert attention, such as visual, auditory, tactile and taste senses. When the warnings used to divert attention are eliminated, attention is focused on the pain again. Different practices, such as kangaroo care, massage, music [16], touch [17], mother’s voice, breast milk and smell [18], sucking [18,19], oral administration of sucrose, glucose or other sweet solutions [20], acupuncture [21], reiki, aromatherapy [22], nesting and fetal positioning [23], are used to control pain. Sensory stimuli are used to activate gate control mechanisms and inhibit nociceptive transmission [14]. There is a need for studies in the literature that support the adoption of evidence-based approaches. This study will contribute to the literature on this topic. It also provides new information by evaluating comfort levels, a parameter other than pain.

### Aim of the Study

According to the literature, it has been reported that newborns’ pain is relieved by stimulating them with olfactory, visual, auditory, tactile, and gustatory senses [24,25]. The sense of smell triggers many motor and emotional responses in the brain [24,25].

At birth, the newborn olfactory system is more developed than other senses. From the first days of birth, newborns can detect and recognize their mother’s body odors and the odors of breast milk and can distinguish different odors, including the smell of breast milk [12,26]. These sensory odor stimuli may block or reduce painkiller delivery by diverting premature infants’ attention from the painful stimulus [24].

When minimally invasive procedures are performed on newborns, the use of non-pharmacological methods for pain control protects newborns from the side effects and possible risks of many pharmacological analgesic agents [19,27,28].

Since a healthy baby is exposed to many painful measures in the first few days after birth, pain control is necessary to mitigate its deleterious effects. Since non-pharmacological methods control the pain with different mechanisms and provide a wide range of treatments, it is necessary to select those methods that have the highest impact on reducing neonatal pain [29]. Within the scope of the neonatal pain control program of the American Academy of Pediatrics and the Canadian Association of Pediatrics, pain assessments are recommended both routinely at regular intervals and before and after painful interventions and that the widespread use of some non-pharmacological practices will positively affect newborn health [30]. Also, The American Academy of Pediatrics also stated that the long-term effects and safety of pharmacological analgesia have not yet been studied [31].

Therefore, this study aimed to evaluate the effect of breast milk smelled on preterm babies’ pain and comfort levels during venous blood collection.

Hypotheses of research

**H1.**  *Breast milk smelled by preterm babies during venous blood collection has a positive effect on pain levels*.

**H2.**  *Breast milk smelled by preterm babies during venous blood collection has a positive effect on comfort levels*.

## 2. Materials and Methods

### 2.1. Type of Research

The research was planned as a randomized controlled experimental study.

### 2.2. Sample

We utilized preterm babies who were hospitalized in the NICU. A priori power analysis was performed on the sample in the G*Power 3.1 program based on the article titled “The Calming Effect of Maternal Breast Milk Odor on Premature Infants” [32]. When the power of the research (1-β) is desired to be 0.80, it was calculated that it was appropriate to include a total of 34 cases in two groups in the sample group. In order to increase the power of the study, a total of 60 (30 experimental and 30 control group) preterm babies were included.

The same inclusion and exclusion criteria were applied for the experimental and control groups:

Criteria for inclusion in the study:

- Being at 28–37 weeks of gestation;
- Not needing oxygen therapy and mechanical ventilation;
- Approval of the neonatal specialist physician of the service to include preterm newborns in the study (verbal approval was obtained from the physician);
- Approval of parents to include preterm newborns in study;
- Not having a serious disease (metabolic disease, congenital anomaly, severe broncho pulmonary dysplasia, NEC) that would prevent the preterm from participating in the study.

Exclusion criteria in the study:

- Not being at 28–37 weeks of gestation;
- Needing oxygen therapy and mechanical ventilation;
- Lack of approval from the neonatologist of the service for inclusion in the study;
- Lack of parental consent for premature newborns to be included in the study;
- Having a serious disease (metabolic disease, congenital anomaly, severe broncho pulmonary dysplasia, NEC) that would prevent the preterm from participating in the study.

The sample information was as follows. The gestational age of 58.3% of the babies included in this study was between 29 and 33 weeks. The average age of babies was determined as 32.70 ± 2.01 weeks. The average postnatal age of the babies was 34.80 ± 1.57 days, and 70% of the babies were 35–37 days old; their average birth weight was 1860.33 ± 481.04 g, and 70% of babies weighed between 870 and 2000 g. The average body weight of the babies on the day they were taken into the study was 1985.67 ± 438.00 g, and the body weight of 55% of the babies on the day they were taken into the study was 1100–2000 g. The average height of the babies at birth was 38.10 ± 3.32 cm. The average height of the babies on the day they were taken into the study was 40.25 ± 2.62 cm. The height of 65% of the babies on the day they were taken into the study was 40–46 cm. The average birth head circumference of the babies was 28.43 ± 1.17 cm. The head circumference of 61% of babies was 29–31 cm. The average head circumference of the babies on the day they were taken into the study was 29.73 ± 1.05 cm. The head circumference of 66.7% of the babies on the day they were taken into the study was 30–32 cm. Of the babies, 56.7% were girls, 58.3% were delivered by cesarean section, 58.3% were hospitalized for 11–50 days, 97.7% were diagnosed with respiratory distress, 46.7% were breastfeeding and 100% were breastfed (Table 1).

Simple randomization method: randomization was performed according to the following order.

(1) Control group: 1, 3, 6, 8, 9, 11, 13, 16, 17, 19, 20, 21, 23, 25, 27, 30, 31, 33, 34, 35, 38, 40, 44, 45, 49, 52, 53, 57, 58, 60.
(2) Experimental group: 2, 4, 5, 7, 10, 12, 14, 15, 18, 22, 24, 26, 28, 29, 32, 36, 37, 39, 41, 42, 43, 46, 47, 48, 50, 51, 54, 55, 56, 59.

### 2.3. Tools Used in the Research

Data Collection Form, Newborn Infant Pain Scale (NIPS), Premature Infant Comfort Scale (PICS) forms were used to collect the data for this study.

#### 2.3.1. Data Collection Form

This form consists of 13 questions and includes date of birth, gestational age, postnatal age, gender, birth weight, birth weight on the day of admission to the study, length at birth, height on the day of admission to the study, head circumference at birth, head circumference on the day of admission to the study, type of birth, day of hospitalization when taken into the study. Information, such as the reason for blood collection, the place where blood was collected, the time of blood collection, NIPS pain score, PBKS score, nutritional content, was collected.

#### 2.3.2. Newborn Infant Pain Scale (NIPS)

It was developed by Lawrence et al. (1993) [33]. Turkish validity and reliability were determined by Akdovan (1999) [34]. It is a pain assessment scale developed for premature babies and newborns and behaviorally evaluates the response to pain in invasive procedures. In the scale, the baby’s facial expression (0–1 point), crying (0–2 points), breathing pattern (0–1 point), arm movements (0–1 point), leg movements (0–1 point) and alertness (0–1 points) are evaluated and scored. The total score in NIPS varies between 0 and 7, scores higher than 3 indicate the presence of pain, and a higher score indicates increased pain severity [33,34,35]. In this study, the Cronbach alpha coefficient for the NIPS scale was 0.89.

#### 2.3.3. Premature Infant Comfort Scale (PICS)

It is a multidimensional scale used to evaluate behavioral and psychological comfort and pain. Turkish validity and reliability were determined by Alemdar and Güdücü Tüfekçi (2015) [36]. It evaluates 7 parameters: Alertness, Calmness/Agitation, Respiratory Status (only in mechanical ventilation support) or Crying (not evaluated because it was scored only in children with spontaneous breathing), Physical Movement, Muscle Tone, Facial Movements and Average Heart Rate. Each item is on a 5-point Likert type, scored from 1 to 5, from worst to best. According to PBKS, the baby’s comfort is evaluated based on the total score. Accordingly, 35 indicates the lowest and 7 indicates the highest comfort score. A high score from the scale indicates that the comfort level is low. If the total score is ≥17, it indicates that a pain-reducing intervention is needed [36]. In this study, the Cronbach alpha coefficient for the NIPS scale was 0.87.

### 2.4. Implementation of the Research

The data in this study were collected by the researcher from preterm babies and their mothers in the NICU via a face-to-face interview method. After the purpose and content of the research were explained to the mothers, data were collected by obtaining their verbal and written consent. The collection process of research data was carried out by the researcher in both groups (experimental and control) in the order specified below.

In order to prevent the mothers from being affected, the baby was made to smell breast milk when the mothers were not present. The data for this study were collected via face-to-face interview with mothers. Before each application, the baby’s physiological/behavioral signs of stress and distress (apnea, SPO2 drop (below 90%), crying, tachycardia, hypotonia, convulsions, facial color change, etc.) were observed. When one of the specified situations was encountered, the process was paused until the values returned to normal levels. Blood collection was performed between 05:30 and 08:30, the routine blood collection time for babies in the NICU. Social hand washing was performed before and after each procedure performed on babies. The application was made during routine blood collection. No blood was taken except what the doctor requested. Routine blood collection (venous) planned by the physician was performed by the nurse providing treatment and care for the baby.

The application was made 30 min before feeding, taking into account the baby’s nutrition and the risk of aspiration. Application time was kept to a maximum of 20 min. In order to avoid ethical violations, the babies in the control group were made to smell breast milk, just like the babies in the experimental group, after all procedures were completed. Smelling breast milk and blood collection process: Care was taken to ensure that the breast milk was freshly expressed or was expressed at least 6 h ago in order to give off a scent. One hour before blood collection, the baby’s mother’s breast milk, which was to be smelled, was taken out of the refrigerator and kept at room temperature for approximately 30 min. Breast milk was placed on a clean breast pad, and each baby’s mother’s breast milk was checked (baby’s surname, breast milk must have been expressed at least 6 h ago). The baby, whose physiological/behavioral parameters were normal, started to smell breast milk before blood collection. The baby’s head was kept in the right/left lateral (side) position for the application. A breast pad moistened with breast milk was placed on the side where the baby’s head is located. This pad was placed 15 cm away from the baby’s head, and the baby began to smell breast milk 30 min before blood was drawn. Blood was taken throughout the blood collection process, and the baby continued to smell breast milk. NIPS and PICS evaluations were made and recorded by the researcher for the experimental and control groups 5 min before, during and 5 min after blood collection.

### 2.5. Analyzing Data

Statistical Package for the Social Sciences (SPSS) 23.0 program was used for statistical analysis of the study data. As descriptive statistics, mean and standard deviation or median and minimum–maximum values were given for continuous variables specified by measurement, and frequency and percentage values were given for qualitative variables. The suitability of continuous variables for normal distribution was evaluated with the Kolmogorov–Smirnov test. Student *t* test was used in independent groups in paired group analyses for parameters with standard distribution. Regression was performed in line with the correlation analysis results of the data. Chi-square test was used for group comparisons of qualitative variables. In all statistical analyses, the significance level was accepted as *p* < 0.05.

### 2.6. Ethical Dimension of Research

Approval was received from the Non-Interventional Research Ethics Committee to conduct the research. In addition, permission was obtained from the institution where my research was conducted. The researchers obtained the necessary consent from the mothers and fathers of the babies they included in the sample. Parents were first informed about the study, and written and verbal consent was obtained from their parents. The research was conducted in accordance with the principles of the Helsinki Declaration.

### 2.7. Limitations

The limitation of this study is that it was conducted in a single center. The data and results collected in the research are limited to the hospital where the research was conducted; therefore, generalizations cannot be made with the results obtained.

Although this study was a randomized controlled trial, there is a possibility of researcher bias during sample selection and scale administration. Therefore, a larger sample size and a fully blinded study are recommended to reduce researcher bias. Furthermore, the study’s power was set at 80, which could have been achieved with a higher sample size. Although two valid and reliable instruments were used in data collection, this study lacked any data that could yield more concrete results. For example, pulse, respiration, and oxygen parameters that change during pain were not assessed.

## 3. Results

We compared the NIPS scores between the two groups. Significant results were obtained in the intervention group. As seen in Table 2, there was no significance in terms of NIPS scores in both groups before the intervention. The NIPS score of the group that was exposed to breast milk during the intervention (1.37 ± 1.04) was statistically significantly lower than the group that was not exposed to breast milk (7.0 ± 3.04) (Cohen’s d: 2.48, CIS: 95) (*p* < 0.05). Then, 5 min after the intervention, the NIPS score of the group that was exposed to breast milk (0.27 ± 0.24.37 ± 1.04) was statistically significantly lower than the group that was not exposed to breast milk (3.43 ± 2.03) (Cohen’s d: 2.18, CIS: 95) (*p* < 0.05) (Table 2).

We compared the PICS scores between the two groups. Significant results were obtained in the intervention group. As seen in Table 3, there was no significance in terms of PICS scores in both groups before the intervention. The PICS score of the group that was exposed to breast milk during the intervention (12.63 ± 11.14) was statistically significantly lower than the group that was not exposed to breast milk (29.37 ± 14.17) (Cohen’s d: 1.31, CIS: 95) (*p* < 0.05). Further, 5 min after the intervention, the PICS score of the group that was exposed to breast milk (9.50 ± 8.89) was statistically significantly lower than the group that was not exposed to breast milk (17.33 ± 18.14) (Cohen’s d: 0,54, CIS: 95) (*p* < 0.05) (Table 3).

When examining the correlation between total NIPS and PICS scores, it was determined that there was a low negative correlation between NIPS and PICS scores before the procedure (r = −0.11, *p* = 0.01). This showed that those with good comfort had lower pain. A strong correlation was found when looking at NIPS and PICS scores measured during the procedure (r = 0.89, *p* = 0.01). This result showed that as the baby’s pain increases, the deterioration in comfort also increases. A strong correlation was found when looking at the NIPS and PICS scores measured 5 min after the procedure (r = 0.86, *p* = 0.01). Accordingly, as pain increased, the deterioration in comfort increased (Table 4).

## 4. Discussion

Multiple studies have declared that breastfeeding affects the response to pain in a sensory–oral way. Also, it has been reported that breastfeeding is one of the natural ways to reduce pain depending on skin-to-skin contact, sucking, and the sweetness of the milk. Recent studies have noted that special and pleasant flavors can reduce pain in neonates. Breast milk (less than 2 mL), along with its proteins, fats, and sweeteners, can effectively lower pain and spontaneous crying in human and mouse infants [12,13,15,37].

It was determined that there was no difference between the groups in terms of NIPS and PICS measurements taken 5 min before the procedure of preterm babies in the experimental and control groups. However, in the measurements made during the procedure and 5 min after the procedure, a significant statistical difference was observed between the groups in terms of NIPS and PICS scoring. According to these results, it was seen that the babies in the group that was exposed to breast milk cried less and were more comfortable. In a study conducted to evaluate the analgesic effects of breast milk scent on newborns, it was determined that newborns exposed to breast milk had lower NIPS scores during intravenous blood collection, shorter crying times, and lower salivary cortisol levels [38]. In another study, premature babies were exposed to their own mother’s breast milk, another mother’s milk, and formula and premature babies’ behavioral pain responses (such as crying, grimacing and motor activity) were evaluated. As a result of this evaluation, it was found that the behavioral pain responses of premature babies were reduced by the smell of their own breast milk, but these reactions were not reduced by the smell of another mother’s breast milk or formula [32]. In studies conducted with newborns at different weeks of age, it was reported that smelling breast milk during different painful procedures had a positive effect on pain [12,39,40]. A study using a NIRS device investigated the effect of sniffing breast milk on pain. In this study, NIRS findings were found to be more positive in babies who were sniffed [41].

It was reported that the smell of breast milk applied during retinopathy examination of preterm babies had a positive effect on the babies’ comfort and pain levels [22,42]. Also, one study found that premature babies who listened to their mother’s voice felt less pain during painful procedures. So, we can say that newborns are born recognizing the smell and voice of their mothers and that they are comforted by the smell and voice of their mothers.

When the correlation between the total NIPS and PICS scores of preterm babies in the experimental and control groups was examined, it was found that there was a strong correlation between the NIPS and PICS scores measured during the procedure and 5 min after the procedure. These results showed that as the baby’s pain increased, the deterioration in comfort simultaneously increased. In other words, it can be said that pain in newborns has a negative effect on the comfort of the newborn and, therefore, on the growth and development of the newborn. It is reported in the literature that newborns who are exposed to painful procedures that disrupt their comfort experience toxic stress and that this stress negatively affects newborn health [43]. In another study where breastfeeding was tried during vaccination, the pain levels of the babies were compared and the pain level was found to be lower in the experimental group [44].

For this reason, it can be said that it may be beneficial for neonatal nurses and other healthcare professionals to use non-pharmacological methods to reduce the pain of all newborns in the NICU. Thus, newborns’ exposure to toxic stress can be reduced and their growth developments can change positively. Many studies have reported that breast milk and breastfeeding have a positive effect on pain [39,40,41,44].

This study supports this conclusion. This study also showed that exposing babies to the smell of breast milk has an impact on their comfort levels.

## 5. Conclusions

At the end of this study, it was found that smelling breast milk, one of the non-pharmacological methods applied, had a significant positive effect on newborns’ ability to cope with pain and their comfort. Smelling breast milk is a method that can be easily applied by healthcare professionals in invasive or non-invasive painful procedures. Additionally, this method is cost-free and has no complications that could harm babies. In line with these advantages, it is recommended that nurses working in the NICU use this application in their daily routine nursing care. In addition, larger samples in multicenter studies are needed for this method to be widely used.

## Figures and Tables

**Table 1:** Demographic characteristics of premature babies.

Data		*n*	%
**Gestational age, mean: 32.70 ± 2.01 week**	29–33 week	35	**58.3**
	34–36 week	25	**41.7**
**Postnatal age, mean: 34.80 ± 1.57**	30–34 day	18	**30.0**
	35–37 day	42	**70.0**
**Birth weight, mean: 1860.33 ± 481.04 g**	870–2000 g	42	**70.0**
	2100–3340 g	18	**30.0**
**Weight when included in the study, mean: 1985.67 ± 438.00 g**	1100–2000 g	33	**55.0**
	2010–3300 g	27	**45.0**
**Height at birth, mean: 38.10 ± 3.32 cm**	27–39 cm	36	**60.0**
	40–43 cm	24	**40.0**
**Height when included in the study, mean: 40.25 ± 2.62**	34–39 cm	21	**35.0**
	40–46 cm	39	**65.0**
**Head circumference at birth, mean: 28.43 ± 1.17 cm**	25–28 cm	23	**38.3**
	29–31 cm	37	**61.7**
**Head circumference when included in the study, mean: 29.73 ± 1.05**	27–29 cm	20	**33.3**
	30–32 cm	40	**66.7**
**Sex**	Woman	34	**56.7**
	Man	26	**43.3**
**Type of birth**	Normal	25	**41.7**
	Cesarean section	35	**58.3**
**The day she stayed in the NICU, mean: 1593 ± 9.86**	1–9 day	25	**41.7**
	11–50 day	35	**58.3**
**Disease**	Respiratory distress	2	**97.7**
	Premature	58	**3.3**
**Mother sucking situation**	Yes	32	**53.3**
	No	28	**46.7**

**Table 2:** Comparison of NIPS scoring (* *t*-test in independent groups, *p* < 0.05).

Measurements	NIPS X ± SD (Experimental Group)	NIPS X ± SD (Control Group)	* t, *p*
**5 min before the procedure**	0.03 ± 0.18 (min: 0, max: 7)	0.01 ± 0.12 (min: 0, max: 7)	t = 1.0, *p* = 0.32
**During the process**	1.37 ± 1.04 (min: 0, max: 7)	7.0 ± 3.04 (min: 0, max: 7)	t = 23.74, *p* = 0.01 Cohen’d: 2.48, CIS: 95
**5 min after the procedure**	0.27 ± 0.24 (min: 0, max: 7)	3.43 ± 2.03 (min: 0, max: 7)	t = 16.20, *p* = 0.01 Cohen’d: 2.18, CIS: 95

**Table 3:** Comparison of PICS scoring (* *t*-test in independent groups, *p* < 0.05).

Measurements	PICS X ± SD (Experimental Group)	PICS X ± SD (Control Group)	* t, *p*
**5 min before the procedure**	7.63 ± 6.68 (min: 7, max: 35)	7.50 ± 6.82 (min: 7, max: 35)	t = 0.76, *p* = 0.44
**During the process**	12.63 ± 11.14 (min: 7, max: 35)	29.37 ± 14.17 (min: 7, max: 35)	t = 15.75, *p* = 0.01 Cohen’d: 1.31, CIS: 95
**5 min after the procedure**	9.50 ± 8.89 (min: 7, max: 35)	17.33 ± 18.14 (min: 7, max: 35)	t = 12.79, *p* = 0.01 Cohen’d: 0.54, CIS: 95

**Table 4:** Examining the correlation between total NIPS and PBCS scores (* Pearson correlation).

Scales PICS	NIPS 5 min Before the Procedure	NIPS During the Process	NIPS 5 min After the Procedure
**5 min before the procedure**	* r = −0.11, *p* = 0.01		
**During the process**		r = 0.89, *p* = 0.01	
**5 min after the procedure**			r = 0.86, *p* = 0.01

## Data Availability

The original contributions presented in the study are included in the article; further inquiries can be directed to the corresponding authors.
